# Use of Doppler ultrasound for non-invasive urodynamic diagnosis

**DOI:** 10.4103/0970-1591.45548

**Published:** 2009

**Authors:** Hideo Ozawa, Toyohiko Watanabe, Katsutoshi Uematsu, Katsumi Sasaki, Miyabi Inoue, Hiromi Kumon

**Affiliations:** Department of Urology, Okayama University, Okayama, Japan

**Keywords:** Bladder outlet obstruction, Doppler ultrasonography, urodynamics, velocity

## Abstract

**Objectives:**

A totally non-invasive transperineal urodynamic technique using Doppler ultrasonography has been developed.

**Methods:**

Since normal urine does not have blood cells, urine was thought not to produce the Doppler effects. However, basic studies confirmed that the decrease of pressure at high velocity (Bernouilli effect) caused dissolved gas to form microbubbles, which are detected by Doppler ultrasonography. Subjects sat and the probe was advanced via remote control to achieve gentle contact with the perineal skin. The digital uroflow data signals and the color Doppler ultrasound video images were processed on a personal computer. The flow-velocity curves from two sites; the distal prostatic urethra just above the external sphincter (V1) and the sphincteric urethra (V2) were plotted against time. The parameters of both the pressure-flow studies and the Doppler ultrasound urodynamic studies were compared in men who had various degrees of obstruction.

**Results:**

Functional cross-sectional area at prostatic urethra (A1), calculated by Q_max_/V1, was lower in the group of bladder outlet obstruction (BOO) vs. control group. Velocity ratio (VR), which was calculated by V1/V2, was the parameter having the best correlation with BOO index, though A1 had a similar correlation. This method is viable to diagnose the degree of BOO.

**Conclusions:**

The development of non-invasive Doppler ultrasound videourodynamics (Doppler UDS) will dramatically expand the information on voiding function.

## INTRODUCTION

Despite much progress in the diagnosis of voiding dysfunction, urologists are often not fully aware of the potential of Doppler ultrasound measurement of urinary velocity. This is probably because ultrasound was thought to give little additional information, except for postvoid residual determination and the size of the prostate gland using the transabdominal approach. Doppler sonography has been widely used for evaluating flow velocities of blood flow. However, no studies have been reported on its application in measurement of urinary flow in the urethra, until our papers have been published.[1,2] Normally urine contains no cells and static urine in the urinary bladder and renal collecting system appears anechogenic.[3] It had been generally assumed that flow without particles could not provide the Doppler effects.[4] In our previous research to develop a non-invasive pressure-flow-like urodynamic evaluation, we demonstrated that both urine and distilled water flowing above a certain flow rate could be detected by Doppler ultrasound due to cavitation of dissolved gases.[1] In the case of fluids containing gases, velocities can be measured accurately by Doppler ultrasonography when the flow exceeds a minimum flow rate. Using the transperineal approach, the urinary stream in the urethra can be clearly detected on the ultrasound monitor and the flow velocity can be measured accurately at any point of the urethra.[2] This method does not need a painful catheterization. Following our papers, Dietz and Clarke compared color Doppler ultrasound with voiding cystourethrogram in 99 incontinent patients. Urine loss could be detected in 56 women by Doppler ultrasonography and on the other hand in 58 patients by x-ray evaluation.[5]

The aim of this review is to evaluate the role of non-invasive transperineal Doppler ultrasound urodynamics, especially for measurement of urinary velocity related parameters in the urethra.

## METHODS (1)

### Basic principle of Doppler ultrasound urodynamic study

Transperineal observation of Doppler ultrasonography

**Figure 1:**
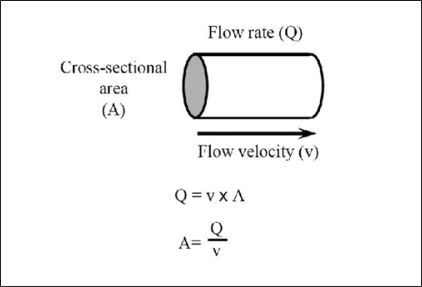
Significance of flow velocity during voiding. Flow rate can be measured at the urethral meatus using a flow meter. Flow rate (Q) divided by flow velocity (v) is assumed to represent the functional cross-sectional area of the urethra (A), which is a direct parameter of obstruction. Obstruction can thus be quantified by cross-sectional area measurement.

To establish the applicability of Doppler ultrasonography as a non-invasive urodynamic technique, we first observed the Doppler image in the male urethra during voiding. Why did we apply Doppler ultrasound? Using Doppler ultrasonography flow velocity can be measured. We can measure the flow rate through the urethral meatus using a flow meter. If we assume that the flow rate divided by the flow velocity represents the functional cross-sectional area of the urethra, which is directly correlated to obstruction, obstruction can be quantified by cross-sectional area measurement [Figure 1]. But the use of Doppler ultrasonography has never been assessed previously with regard to velocity measurement in the urethra. Generally, it has been assumed that flow without particles could never provide the Doppler effects. During normal Doppler ultrasonography, blood cells are playing a great role in producing the Doppler effects.[6] Since normal urine does not have blood cells, urine was thought not to produce the Doppler effect. However, we demonstrated practically that this assumption is false.

The practical observation of the ureteric jet effect, the echographic appearance of urine entering the bladder has been well known.[7] We presumed that urinary flow in the urethra would also be visible using Doppler ultrasonography. When we tried to attach the ultrasound probe at the perineal region, the urinary stream in the male urethra could be clearly detected. Why Doppler signals can be obtained from urine without particles? Microbubble formation in a high flow state might present a possible explanation. We therefore conducted basic experiments.

### Artificial urethral model study

A cellulose tube as a collapsible tube was inserted into an ultrasound gel pad to form an experimental urethra [Figure 2a]. Three different fluids, degassed water, urine, and liposome (multi-lamellar particles) solutions were used to compare signal intensity and flow velocity at various flow rates. The flow rate from the tube was measured with an uroflowmeter. The Doppler image and digital uroflow signal data were processed by a computer.

**Figure 2a:**
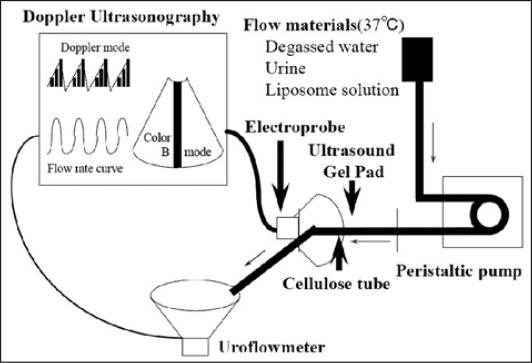
Diagram of the experimental model. A 3.75 MHz micro-convex electroprobe was used for color Doppler ultrasound. The flow rate of the peristaltic pump was set from 0.3 ml/s to 4.0 ml/s. A cellulose tube, 30 cm in length and 6 mm in internal caliber was inserted into an ultrasound gel pad so that its proximal 10 cm was presented within the pad. The ultrasound probe was positioned opposite to the direction of the flow at the distal boundary of the ultrasound gel pad.

### Results (1)

Sufficient Doppler signals and flow velocity curves could be obtained in the cases of urine and liposome solution, while degassed water showed no Doppler signals at any flow rate tested [Figure 2b]. The minimum flow rate, at which clear Doppler signals were continuously detected in the frontal plane, ranged from >1.5 ml/s for urine, and >0.3 ml/s for liposome solution. The maximum flow velocities were identical in these two materials at a flow rate >2.0 ml/s. The functional cross-sectional area of the tube showed a constant value irrespective of the initial flow rate. These results suggest that flow velocity can be measured by Doppler ultrasound above a certain minimal flow rate. Dissolved gases play an important role in creating these Doppler signals. We hypothesize that microbubbles created in the liquid based on Bernoulli's principle and/or tiny particles in the urine during flow are responsible for the Doppler effects. Bernoulli's principle states that when the velocity of a fluid is high, the pressure of the flow becomes low, and when the velocity is low, the pressure becomes high.[8] In general, dissolved gases will form microbubbles by a decrease in pressure or increase in temperature.[9] Since the temperature is stable in our experimental model, decreased pressure due to a high velocity must cause the dissolved gasses to form microbubbles, which enables the Doppler effect. This idea of microbubble formation at high flow rates might also explain the observation of the ureteric jet effect.[7]

**Figure 2b:**
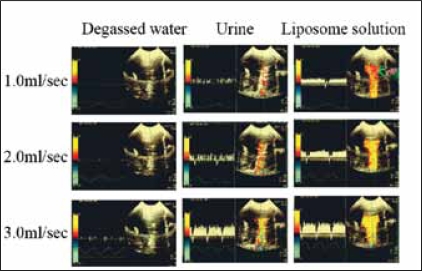
Comparison of color Doppler signals among four flow materials. With degassed water Doppler signals could not be detected on the display at any flow rate (0.3-4.0 ml/s). At 1.0 ml/s no Doppler signal was detected from distilled water or urine. Continuous flow signals were detected using the liposome solution at flow rate 0.3 ml/s or higher. At 2.0 ml/s flow rate, continuous flow signals were obtained from urine and liposome solution

The presence of stenosis with a complicated shape would produce inhomogeneous flow that would contribute to cavitation, and the above-mentioned flow velocity should represent the mean velocity of complex flow through the stenosis. Liquid in a tube shows laminar or turbulent flow, and the type of flow is determined by Reynolds number (R) where R = ρVD/μ (V=flow velocity, D=inside diameter, ρ=specific gravity, μ=viscosity). A high Reynolds number (R > 2100) indicates turbulent flow, while a low Reynolds number (R<2000) indicates laminar flow.[10] Blood flow is usually laminar, mainly due to low viscosity, urine is likely to show turbulent flow in the urethra, which increases fluctuation of the regional flow velocity and flow pressure. Interactions between such factors inside a collapsible tube may explain why a smaller pressure decrease was required to create Doppler signals in the urethra.[10]

### Comment

Our basic studies using an experimental urethra confirmed that the decrease of pressure at a high velocity caused dissolved gas in the urine to form microbubbles, which are detected by Doppler ultrasonography.[1]

## METHODS (2)

### Transperineal approach

Real time ultrasound is an exciting and potentially valuable tool enhancing clinical practice. For evaluation of lower tract dysfunction the use of transabdominal ultrasonography was first documented in the early 1980's, with transrectal and transperineal technique developing somewhat later. We have been conducting a clinical investigation on the applicability of transperineal Doppler sonography to the measurement of flow velocity in the male urethra. For this purpose, we have developed a specially devised robotic probe holder, which can be controlled remotely to obtain an angle in the frontal plane of the urinary stream in the urethra [Figure 3a]. Doppler ultrasound has never been used previously for velocity measurement of the urinary flow in the urethra. For previous investigators probably the difficulty in aiming the ultrasound probe at the urethra to get an accurate visualization of the proximal urethra was a major hurdle. A transabdominal approach cannot adequately visualize the urethra during voiding. Although transrectal approaches are frequently used for the diagnosis of prostatic diseases, direct compression of the prostatic urethra is unavoidable during voiding. Moreover, these two approaches do not provide an angle facing the frontal plane of the urinary flow, which is the angle most suitable in detecting Doppler effects. The transperineal approach offers the ideal angle of detecting Doppler effects in the bladder neck and prostatic urethra.[2] Since the image in this approach was limited to the mid-sagittal plane, we developed specially equipped robotic arm device. So far B-mode pelvic floor imaging has been limited to mid-sagittal view. With the introduction of the Doppler imaging we are now able to access dynamic velocity measurement of voiding.

**Figure 3a:**
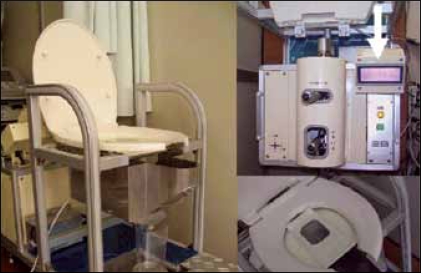
A specially devised perineal ultrasound probe robotic holder. Subjects sat on an uroflow micturition chair. The ultrasound probe was covered with solid echogel and advanced via remote control to achieve gentle contact with the patient's perineal skin, manipulating levers using the position indicator. Arrows indicate the position indicator.

### Doppler ultrasound videourodynamic system (Doppler UDS)

#### Equipment

We used an ultrasonic image-directed color Doppler system (SSD-5500, Aloka), with a 3.75 MHz micro-convex electroprobe for this study. The probe holder was remotely controlled by a robotic arm to obtain a frontal plane angle of the urethral urinary stream. Imaging can be performed in sitting position. The ultrasound probe was covered with solid echogel and advanced via remote control to achieve gentle contact with the patient's perineal skin. Bladder filling should be specified. The presence of a full rectum may impair diagnostic accuracy and sometimes necessitates a repeat assessment after bowel emptying. The standard mid-sagittal field of vision includes the symphysis pubis anteriorly, the urethra and bladder neck, the prostate gland, seminal vesicles, rectum, and anal canal. Natural micturition was observed under ultrasound monitoring. Uroflow rates from the urethral meatus were measured with an uroflowmeter (Urocap-2: Laborie Medical Technology Company: Canada). This prototype system was designed to measure both flow velocity in the urethra by Doppler ultrasound and flow rate by uroflowmetry simultaneously and non-invasively [Figure 3b]. The digital uroflow data signals and the color Doppler ultrasound video images were processed on a personal computer.

**Figure 3b:**
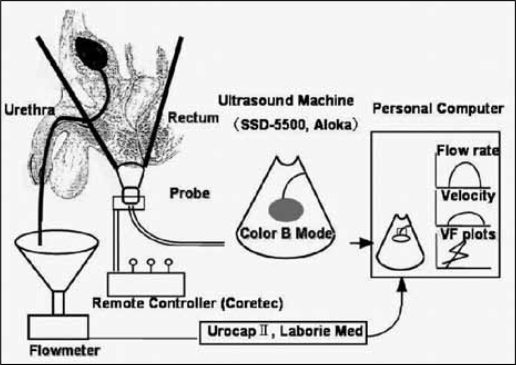
Diagram of the ultrasound urodynamic equipment setup. The ultrasound probe was operated by specially equipped remote controlled robotic device. The color Doppler flow image, flow velocity curve, and flow rate curve were displayed on the computer monitor.

### Data processing

Information on Doppler shift frequencies derived from different flow velocities was converted to color encoded data using the system software of SSD-3500. These color scale data were recorded at intervals of 0.9 s, then analyzed by custom software (API-TOOL, Contec) running on a personal computer (GL5133, Celebris) using Microsoft Windows. Multiple rectangular areas of any size could be set at any point around the bladder and the urethra. The color-coded data of each 9 pixels/mm2 in the sample area were averaged to reduce technical artifacts. This calculation was performed every 0.9 s and a flow velocity curve in the region of interest was obtained. The Doppler angle, the angle between the direction of flow and the direction of sound propagation, was also measured and angle correction was performed to detect each flow velocity vector.

**Figure 4:**
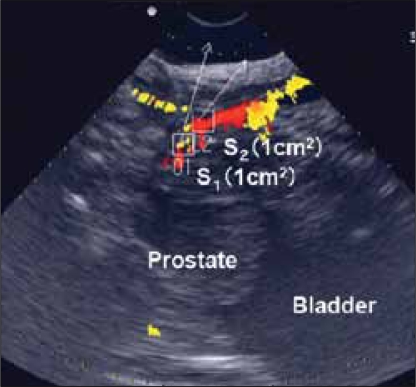
Schematic overview of parameters. V1 is the velocity at the distal prostatic urethra (S1); V2 is the velocity at the membraneous urethra (S2); A1 is the functional cross sectional area at S1, which equals Q/V1; A2 is the functional cross sectional area at S1, which equals Q/V1; and VR is the velocity ratio, which equals V1/V2.

The flow-velocity curves from two sites; the distal prostatic urethra just above the external sphincter (S1) and the sphincteric urethra (S2) were plotted against time. The maximum flow velocities at both sites (V1 representing the velocity at S1, V2 that at S2) were recorded at the same instant. From these data, the velocity ratio (VR=V1/V2), and the functional cross-sectional area at S1 (A1) were computed using the formula: A1=Q/V1 [Figure 4].[11]

### Comparison to conventional pressure-flow study

Urodynamic pressure-flow studies (PFS) are accepted by most authorities as the most accurate and important test for the diagnosis of bladder outlet obstruction (BOO).[12,13] However, the main disadvantage of PFS is the need for catheterization. Catheterization causes partial obstruction during micturition, and confers the undesirable consequences of introducing infection and causing discomfort that may alter the micturition reflex. Many patients simply cannot void during a pressure-flow study.[14,15] Attempts have been made to perform PFS using a temporary suprapubic catheter. This invasive procedure increases cost, pain to the patient, and the risk of injury to the bladder and adjacent organs. A suprapubic tube also alters bladder sensation and can alter the normal micturition reflex. In an effort to circumvent these problems, the development of a non-invasive but accurate urodynamic method of diagnosing BOO has been the goal of urodynamic experts for many years. We have been conducting a clinical investigation to develop accurate Doppler ultrasound urodynamic parameters that correlate best with the urethral resistance measured by PFS.

The same investigator conducted both PFS according to the International Continence Society Guidelines and the Doppler ultrasound urodynamics using the above-mentioned method. Urethral resistance was quantified using the BOO index (BOOI),[16] according to the following formula: BOOI = Pdet.Q_max_ - 2×Q_max_, where Pdet.Q_max_ is the detrusor pressure at the point of maximum flow rate and Q_max_ is the maximum flow rate using PFS. Based on the BOOI, bladders were categorized as obstructed (BOOI more than 40), equivocal (20 to 40), or unobstructed (less than 20).

### Results (2)

**Figure 5:**
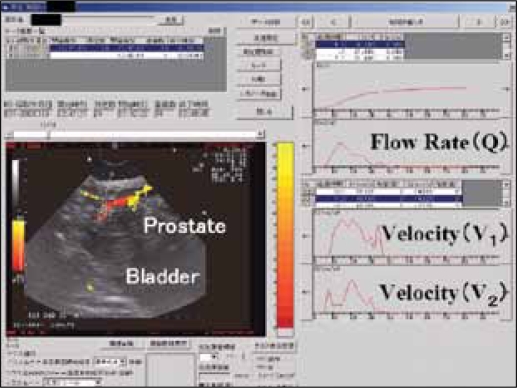
Voiding analysis of a case with benign prostatic enlargement: Left lower portion: A retrieved picture obtained from Doppler ultrasound, which demonstrated an enlarged prostatic nodule. Note that although the distal prostatic urethra was clearly identified from the prostatic nodule, the proximal site of the prostatic urethra did not show a Doppler signal. Right lower portion: Flow velocity curve obtained by Doppler ultrasound. Right upper portion: Flow rate curve obtained by uroflowmetry.

Figure 5 illustrates the Doppler ultrasound urodynamics of a case with benign prostatic enlargement. The left lower portion demonstrates an enlarged prostatic nodule. The right upper portion shows a flow rate curve. The right lower portion shows the flow velocity curve at the prostatic urethra, which was obtained from the Doppler ultrasonography.

The parameters of both the pressure-flow study and the Doppler ultrasound urodynamic studies were compared in men who had various degrees of obstruction (n=22). VR at maximum flow rate was the parameter having the best correlation with BOOI (rho=0.728: P<0.001), though A1 had a similar correlation (rho=-0.708: *P*=0.001). All men with VR exceeding 1.6 were obstructed by pressure-flow criteria (BOOI more than 40). Similarly, all men with VR below 1.0 were equivocal or unobstructed (BOOI 40 or less). This means that although flow was accelerated through the sphincter in the unobstructed group and equivocal group, flow velocity was reduced through the sphincter in the obstructed group.[17] VR and A1 were found to be the best parameters for diagnosis of BOO. The reliability of this method was subsequently validated by Ding *et al*., the retest correlation using Spearman's rho for VR in terms of intra-rater and inter-rater reliability was 0.95 and 0.57, respectively; that for A1 was 0.97 and 0.64, respectively.[18] In a recent report by Nose *et al*. (2005), this noninvasive urodynamic method has a better correlation with obstruction than the other noninvasive method using transabdominal ultrasound of the intravesical prostatic protrusion (IPP).[19,20] We found a non-invasive pressure-flow like urodynamic evaluation based on Doppler ultrasound was viable to diagnose and localize BOO with reasonable reliability.

## CONCLUSION

The development of non-invasive Doppler ultrasound videourodynamics (Doppler UDS) will dramatically expand the information on voiding function. Patients only have to sit and void without painful catheterization. Although some of the analytical methods and the robotic arm related technology still need to be confirmed by larger-sample studies, these applications will become more attractive to the neurourologist. Repeated innovations in associated technology will further broaden the applications of Doppler UDS and make them more informative. The current revolution in computer engineering, robotic technology, imaging, and information technology will advance Doppler UDS within the next decade.
